# Supplementary material for: Reprogramming metabolic pathways in vivo with CRISPR/Cas9 genome editing to treat hereditary tyrosinaemia
Source: Nat Commun. 2016 Aug 30;7:12642. doi: 10.1038/ncomms12642 (PMC5013601; doi:10.1038/ncomms12642)
Supplement: Supplementary Information — Supplementary figures 1-4 and Supplementary Tables 1-3 [file ncomms12642-s1.pdf]

CTGGAGCAGAAATACCAGCTCTCAGTCTC**AGGGGGAGGACAAGGTATCG**ACACAGGAAA
AATGTCAGTAGATGTGAGACACTGATGCCTGCAAACTCAAATTTGTCCCTGCTGTCCCC
TCCCGTTGATGGAGCGGAAGTCAATCTTCTTCCTGAAGGAGCCCCTTCTTAAATGGTTG
CCAATCTCTTCTTAAACCTGGTCAAGCTGGGCAGTGGTGGCACACGACTTTAATCCCAG
CACTTGGGAGGCAGAGGCAGGTAGACTTCTGAGTTCGAGGCCAGCCTGGTCTACAGAGT
GAGTTCCAGGACAGCCAGGGCTACACAGAGAAACCCTGTCTCGAAAAACCAAAAACAAA
AAACAAAAAACTGTGGTCACCCATACTGTTCTCACGTAAGCTTATTTCCTTCCATGACC
CCTGGGAAGGCCTTCTGTGGGGAATTTCCCAATTCTGATCCTTCAAGGGGCCCAAATCC
TAGAACAAGAAATGTCTTAGGAGGTTAGCCAAAGATGGGAGCAGGGTGAGTCCCATTCT
CGGAGGTTTTGGGCTTGAGGCTGAGGTGGGGTGGCCAAGGTAACCTGCTTCCTGGGACT
CATCCTCCTCGTTCCTCCCTCATGCAG**CCTGAGAGAGGCCGGTTCCTCCATTTCCACTC**
GGTGACCTTCTGGGTTGGCAATGCCAAGCAGGTAGAGAGGCTGGACGTAAGGGGCTGTG
GGGGTTGGGGGAGGGCAGAGGACAAGCCAAAGGGCCTCCTTAAGGGGGCTGGACCCAGC
TGGAACTGGGATTGGCTAGTGCGAGGGGAGGATCCAGGCCAGGCTAGGACTGGCACTTT
GGAACCTTGTTTTGCAATCAGGGTCCCCAAGGACCTTGCAGAGGGAGGGGGACAGCTGC
CTTTCTTCCTGGGAGAGTCTCCAAATGACGGACATGGGGGCAGTCAGGAGACTGGGGCT
CCCTTCTAGGGCTCTCCAGACTGGTCCCCACAACCCCCTGGCCCCCCTGCCGCTTTGTA
TACTTTCCAG**GCTGCTTCCTTCTACTGCAACAAGATGGGCTTTGAACCTCTGGCCTACA**
GGGGCCTAGAGACTGGCTCCCGGGAGGTAGTCAGCCACGTCATCAAGCAAGGGAAAGTG
AGTACACATCCAGGTGGCTTGGTGATGAGGGCACCAGTGTGTCCCGGATGGATGTGTG
ATTGTGTCAGGACACATACCTAGCTGGTTCCAAGATGTATGGCTAAGCTGCAACTGACC
CATGTCCCTCCCAGGCCACAGGAATCTTCCTGGTTTCAAACTTCTCAAACTCACCCAGC
ACCACCTATAGAAACTCTAGGAAACTAGGATGTAGGGAAGGGCTTTTCAGTG**GTCTCTG**
GTCCATGGGGTTGCTGGAGTCAGCTAGGCACCAGCCACTGAGAACCCAGTTGGTGGATA
TAGGGGA

Blue- primer   Red- gRNA targeting sequence   **Bold**- Exons

Supplementary Fig 1 | Genomic region of the *Hpd* gene targeted for editing by CRISPR/ Cas9. gRNA's (red) were designed at least 100 bp away from target exons (bold). The primers used in the PCR reactions for the detection of deletions are highlighted in blue.

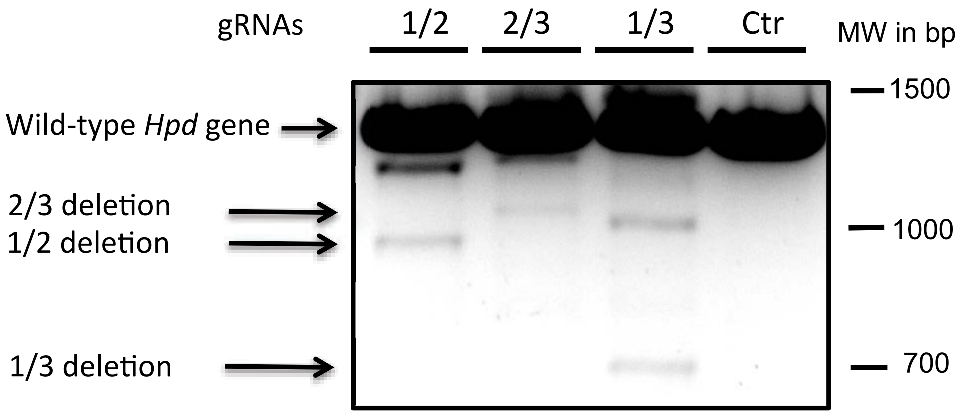

Supplementary Fig 2 | Validation exon excision approach using gRNAs targeting the *hpd* gene. NIH 3T3 cells were transfected with Cas9 only (Ctr: control) or in addition with each gRNA pair as indicated. Deletions of the intervening exons can be detected by PCR (indicated bandshifts) 48 hours post-transfection. MW; molecular weight given in base pairs (bp)

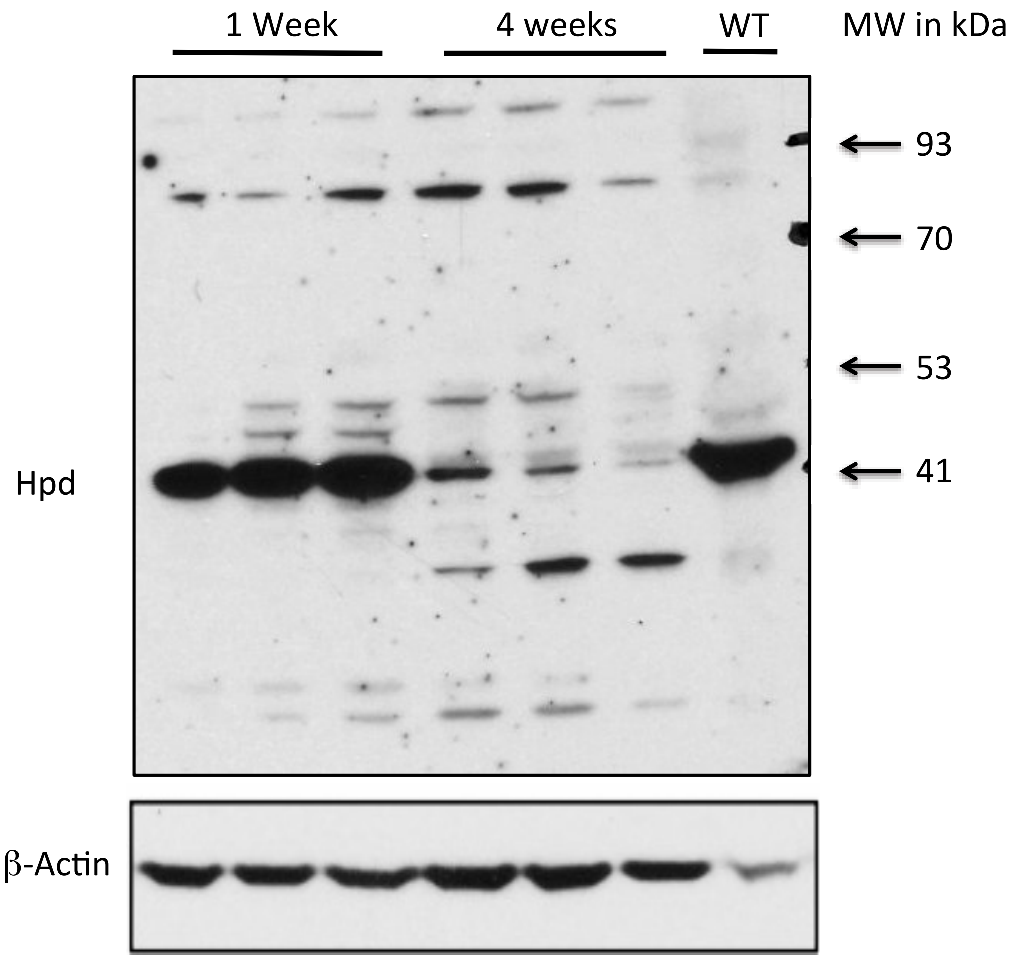

Supplementary Fig 3 | Reduction of HPD protein in mice treated with Cas9 and gRNA1/3. Western blotting of liver homogenate shows a reduction of full-length HPD protein and small amounts of a truncated protein after 4 weeks. The size of this new band corresponds to the HPD protein without exons 3 and 4 (deletion gRNA1/3). MW; molecular weight given in kilo Dalton (kDa)

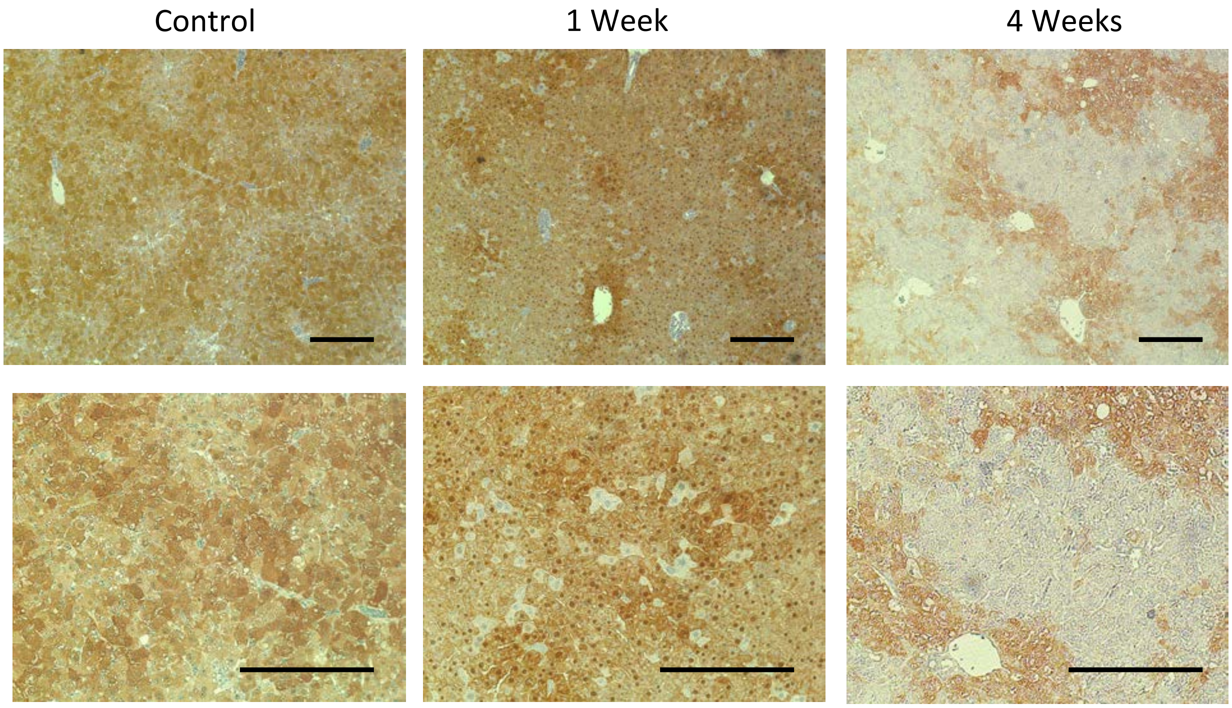

Supplementary Fig 4 | Immunostaining for HPD in mice treated with Cas9 and gRNA1/3 targeting the *Hpd* gene. 1 week after injection, a few single cells do not express HPD and 4 weeks later, the majority of hepatocytes are HPD negative. Control is Cas9 only injected mice. Bar scale is 50 μm.

Name	Sequence	Mismatches	Gene (Exonic)	Location	Measured off-target [% reads]
sgRNA 1	AGCCCAAAACCTCCGAGAAT GGG			chr5:-123632003	
OT1-1	TTCCAAAAACCTCCGAGAAG AGG	4MMs [1:2:5:20]		chr5:-65742503	0.177
OT1-2	AGCCCAAATTCACCGAGAAT TAG	3MMs [9:10:12]		chr8:-115280394	0.028
OT1-3	AGAACAACACCTCCGAAAAT GGG	4MMs [3:4:8:17]	NM_207239(Gtf3c1)	chr7:-132806144	0.332
OT1-4	GGCCCAAACCCTCCAAGAAT GAG	3MMs [1:9:15]		chr7:+87955093	0.099
OT1-5	AGCCTGGAAGCTCCGAGAAT TAG	4MMs [5:6:7:10]		chr16:+35193363	0.071
OT1-6	AGCTAGAACCCTCCGAGAAT CGG	4MMs [4:5:6:9]		chr5:-126246830	0.219
OT1-7	AGTCTAAATCATCCGAGAAT GGG	4MMs [3:5:9:11]		chr6:+67176608	0.000
OT1-8	AGACAAAAGCGTCCGAGAAT AGG	4MMs [3:5:9:11]		chr2:-127979956	0.198
OT1-9	ACCATAAAACCTCCAAGAAT AAG	4MMs [2:4:5:15]		chr8:+55239787	0.155
OT1-10	AGCGCCAAAGCACCGAGAAT GAG	4MMs [4:6:10:12]		chr2:-115591157	0.000
sgRNA 2	AGTCTCCAAATGACGGACAT GGG			chr5:-123632003	
OT2-1	TCTCTCCTGATGACGGACAT TGG	4MMs [1:2:8:9]		chr3:-100413679	0.017
OT2-2	AATATCCAAATGACAGACAT GAG	3MMs [2:4:15]		chr19:+46455960	0.026
OT2-3	ATTCCCCAGCTGACGGACAT TGG	4MMs [2:5:9:10]		chr19:-4462060	0.080
OT2-4	ACTGGCCAAATGACGGACAC TGG	4MMs [2:4:5:20]		chr13:-97760330	0.046
OT2-5	GCTCCCCAAATGACGGGCAT GAG	4MMs [1:2:5:17]		chr10:-86694737	0.011
OT2-6	TGTCTCTGAATTACGGACAT GAG	4MMs [1:7:8:12]		chr4:-66831498	0.026
OT2-7	AGTCACCTAATGAAGGACAT GGG	3MMs [5:8:14]		chr3:-93363331	0.043
OT2-8	AGTGTCCTAGTGACAGACAT GAG	4MMs [4:8:10:15]		chr17:-9603894	0.063
OT2-9	AGTCACCCACTGACTGACAT TAG	4MMs [5:8:10:15]		chrX:-50438406	0.063
OT2-10	TGTCTCCACATGACGAACAT AAG	3MMs [1:9:16]		chr15:-58589519	0.072
sgRNA 3	TGCAGCTTAGCCATACATCT TGG			chr5:-123632003	
OT3-1	TTCAGCTTGGTCATACATCT GAG	3MMs [2:9:11]		chr15:-93971663	0.000
OT3-2	CGCACATTAACCATACATCT TAG	4MMs [1:5:6:10]		chr11:+33532084	0.054
OT3-3	AGCATGTTACCCATACATCT CAG	4MMs [1:5:6:10]		chr1:+128544662	0.069
OT3-4	AGCAGCTGAAACATACATCT GAG	4MMs [1:8:10:11]		chr4:+57698082	0.040
OT3-5	TGAAGCTGAGCCATACAGCT CGG	3MMs [3:8:18]		chr7:-30215494	2.819
OT3-6	TGCCTACTAGCCATACATCT AAG	4MMs [4:5:6:7]		chr4:+120838831	0.046
OT3-7	TGCAGCCTGGCCATACATCA GAG	3MMs [7:9:20]	NM_026183(Slc47a1)	chr11:+61181249	0.072
OT3-8	TGATGCTTAGCCATAGATCT AGG	3MMs [3:4:16]		chr15:-77059088	0.046
OT3-9	TGAATCTGAGCCATACATTT AGG	4MMs [3:5:8:19]		chr5:-29016063	0.043
OT3-10	TCCAGCCAAGCAATACATCT TAG	4MMs [2:7:8:12]		chr1:+155079641	0.040

Supplementary Table 1 | Predicted off-target cutting sites and measured frequencies. The top 10 off-target sites for each gRNA were predicted by the online software (crispr.mit.edu). The sites were ranked based on composite scores, which are generated based on the number and relative location of mismatches between the gRNA and the indicated chromosomal region. Actual off-target cutting was determined by deep sequencing of off-target PCR libraries (see methods) one week after transfection.

gRNA pair	Cut rate	Inverted	Uncut rate
gRNA 1/2 week 1	19.30%	0.02%	80.68%
gRNA 1/2 week 4	61.75%	0.13%	38.12%
gRNA 2/3 week 1	15.16%	0.01%	84.83%
gRNA 2/3 week 4	24.22%	0.07%	75.72%
gRNA 1/3 week 1	15.41%	0.04%	84.55%
gRNA 1/3 week 4	63.18%	0.34%	36.48%

Supplementary Table 2 | Frequencies of *Hpd* deletion and genetic inversion of selected gRNA pairs. Frequencies were determined by deep sequencing of *hpd* libraries (methods) 1 and 4 weeks after transfection of the murine liver.

primer name	sequencing primer
**on-target (HPD)**	
mHPD_For3	GTCTTAGCGGTCTCCCTGTG
mHPD_Rev3	GTCAGCGGAACTTGATGTGG
**off target**	
**sgRNA 1**	
OT1-1_Forward	TGATTCAGTTAGGGTTGGGGC
OT1-1_Reverse	GGAAAACAAACTCAGGTCGGC
OT1-2_Forward	AGTCCCGCCTTATTCCAGGT
OT1-2_Reverse	ACTGATGGCTTCCCTACCCA
OT1-3_Forward	AGGTTCCCACTGCAGCTTTC
OT1-3_Reverse	TGAATGCCAGCAACACAAACC
OT1-4_Forward	GAGGAAGAAAGCCCACCCAG
OT1-4_Reverse	CCAGGACAGCTAGGACTGGT
OT1-5_Forward	CCGGTCTCCTTTGCCCTTAG
OT1-5_Reverse	GCCACCTTCAAGTAGACCCA
OT1-6_Forward	TACACCCATCTTCTCTGCACG
OT1-6_Reverse	CACCATTTGACCCTCTGCCC
OT1-7_Forward	TTTCAGGACAAAGCGCCAAG
OT1-7_Reverse	CTTCGGGGCTTCTCTCAGAC
OT1-8_Forward	GGCATGTGACACCTAGGACC
OT1-8_Reverse	GGTAGATCTGTGACGACCGC
OT1-9_Forward	AGGCACATCTCCGTCACTCT
OT1-9_Reverse	TCACTGAACCAAAAGGAGCAC
OT1-10_Forward	TTCACTTCAGGCTGTCACGC
OT1-10_Reverse	ATTTGGGGGTGTTGTAGGGG
**sgRNA 2**	
OT2-1_Forward	TGAGATGGATGTGTGAGTGGC
OT2-1_Reverse	GCTTCGGTGATGCAACTGTC
OT2-2_Forward	AAACAGCTCCTTTCCCACGA
OT2-2_Reverse	CAAGCCTCCACATACACCCC
OT2-3_Forward	GCTTTGTGTGATGCTGGGGA
OT2-3_Reverse	CTTGTGTCACACGCCATCCT
OT2-4_Forward	GATTTCTGCCCACTCCTCCC
OT2-4_Reverse	TTAGTGAGAAGTGTGCGGGC
OT2-5_Forward	ATGCATGCACTCAAAAACAGGA
OT2-5_Reverse	AGGCTTGGATGTGGGAAGTT
OT2-6_Forward	GTCCCATCCTCATCACAGGC
OT2-6_Reverse	CAGAGGGGAATAATGTGCCAG
OT2-7_Forward	TGCAATGAGAGACCAAGGGC
OT2-7_Reverse	CCAACTCTCAGACCAGTGCC

OT2-8_Forward	GAGTGTCAGGTAGCCTTGGT
OT2-8_Reverse	GCTGCAGGTTTGGGGATGAT
OT2-9_Forward	CTGCCATTGTGTGCCAAGAG
OT2-9_Reverse	ATGGACCCAGGAACATGTGAG
OT2-10_Forward	CAGTCAGGTCCCAGTGTAGC
OT2-10_Reverse	CATGCCTGACACTCTCTGCC
**sgRNA 3**	
OT3-1_Forward	AACTCTTTGCCAGCCCAGAC
OT3-1_Reverse	AGGAGTGTGTGGGTTGGTTG
OT3-2_Forward	GAGATTTGCCTATCCCTGCCT
OT3-2_Reverse	AGATCTCGGGACCAGGGAAG
OT3-3_Forward	TGAAATTGCAGTGGTCCTCC
OT3-3_Reverse	GAGGAAAGGATGGTGTGAGGG
OT3-4_Forward	TGCGTTAGTTCTCCACAGCG
OT3-4_Reverse	CAATGTGTAGAGGTGCCTGC
OT3-5_Forward	CCGAATTAGCCCACACCCTT
OT3-5_Reverse	TGCCCTGAACTTGTGGTGAT
OT3-6_Forward	GCACAGGAGGGACAAATGTT
OT3-6_Reverse	TGCATTCTGGGGTGAAGAGT
OT3-7_Forward	CTCTACCCTCTCCCATGCCT
OT3-7_Reverse	TACCCTCCAGCCCTATTCCA
OT3-8_Forward	CTCGGTGCCTTGTGATTTGG
OT3-8_Reverse	AATGCCACCCAGTTAGTCCAG
OT3-9_Forward	GAAGCTGCTCCCTCCTATCG
OT3-9_Reverse	TATGCAAGGTCCTGGGTTCC
OT3-10_Forward	TCTGAAAGCACGATGGACCT
OT3-10_Reverse	TCCCACGGCTACTTTCCATTG

Supplementary Table 3 | Primers for deep sequencing of on- and off-target sites.
